# Teacher Training in Chile: Where Are Universities Looking? A Narrative Review

**DOI:** 10.3390/ijerph191912802

**Published:** 2022-10-06

**Authors:** Antonio Castillo-Paredes, Karen Núñez-Valdés, Cristian Villegas Dianta, Neliot Villena Olivares, Marisol López Núñez, Mario Fuentes-Rubio, Gerson Núñez-Valdés

**Affiliations:** 1 Grupo AFySE, Investigación en Actividad Física y Salud Escolar, Escuela de Pedagogía en Educación Física, Facultad de Educación, Universidad de Las Américas, Santiago 8370040, Chile; 2 School of Education, Faculty of Education, Universidad de Las Américas, Santiago 8370040, Chile; 3 School of Pedagogy in Physical Education, Faculty of Education, Universidad de Las Américas, Santiago 8370040, Chile; 4 Department of English, Faculty of Education and Social Sciences, Universidad Andrés Bello, Santiago 8370134, Chile

**Keywords:** teacher, diagnostic, evaluation, training, university

## Abstract

Initial Teacher Training (ITT) has been a recurring theme in recent years in Chilean educational policy and the field of educational research, mainly due to its impact on the Chilean educational system. Under its relevance, this article analyzes ITT and some aspects of its improvement through the revision methodology described by Kart and Kart (2021). Among the main findings, it is possible to mention that the research provides evidence on the decisions that should be made around the teaching profession and the improvement of initial training not only at the institutional level but also at the educational policy level. At the same time, it highlights the need for comprehensive support for future pedagogues and the importance of using the data obtained through the ‘National Diagnostic Evaluation’ for decision making.

## 1. Introduction

In 1998 the need to strengthen the fundamental functions of higher education systems was mentioned at the UNESCO World Higher Education Conference [1]. Among many of its contributions, the improvement of society was highlighted. Likewise, the Tuning Project pointed out the importance of constantly verifying the demands of society, emphasizing interpersonal skills and the social commitment that future professionals in training must develop [2,3].

In this sense, higher education must ensure that it contributes to social justice [4], considering the concept of University Social Responsibility. Thus, institutions must seek an organizational model that contributes to society [5]. In higher education institutions, the teaching staff must adjust their pedagogical proposals based on the guidelines declared by the university [6]. Then, students would acquire skills that will allow them to behave professionally with a sense of social responsibility [7,8]

It is considered that higher education must be of quality. However, this is a polysemic concept with different approaches to it. Acevedo et al. [9], in their systematic search, declare that the approaches to determine quality are ‘fitness for purpose’ and ‘value for money’. Their development presents a neoliberal vision based on accountability as quality control. According to Perez-Juste [10], quality education aims to educate people by providing the tools to perform successfully in various circumstances whilst overcoming them.

This research develops around the field of higher education in Chile. Different types of certificates are awarded at this level, depending on the institution’s nature. This could be ‘Professional Institutes’ and ‘Technical Training Centers’ that award college certificates or diplomas. Universities issue professional degrees and are categorized as private and public institutions depending on their funding [11]. Act 20,903 establishes that only accredited universities (public or private) can grant a teaching degree to ensure the quality of teacher training.

The implementation of this act has decreased the enrollment in teaching degrees in Chile. Nevertheless, this has mainly affected private universities. Furthermore, the enrollment decrease has been exacerbated by the increase in the score requirements for admission to this type of degree [12].

To generate a homogeneous basis in ITT and to establish quality criteria, the Ministry of Education has developed the Teacher Training Standards for every teaching degree [13]. This is closely related to the need for high-quality training for Chilean teachers. Under the logic of quality education, universities have also accepted the call to contribute to it. The United Nations [14] has generated the so-called Sustainable Development Goals [15].

Goal number four refers to the need to establish quality education, which is evidenced, among other things, in the increased number of scientific publications [16]. In this context, teacher training plays a key role in the development of societies facing the 21st century [17]. Therefore, this paper aims to answer the following questions: How do Chilean universities approach ITT? Do universities consider student support and guidance fundamental? What role do the data obtained from the National Diagnostic Evaluation have? What approach have researchers adopted regarding this issue?

### Theoretical Framework

There is concern about the quality of teacher training within the Chilean educational system, which is evident in the policies and measures aimed at increasing its quality [18]. One of the most critical measures in this direction is the creation of the ‘Initial Program’ (Programa Inicia in Spanish), which since 2008 has incorporated three components: a definition of guiding standards for teaching degrees, university support programs, and an evaluation at the end of professional training (called Initial Test). The evaluation focused on assessing students’ performance at the end of their university training [19]. However, since the results of this evaluation have not been encouraging, minimum scores have been established to study any teaching degree to increase selectivity through Act 20,903 on the Teacher Professional Development System. In addition, the Initial Test was replaced by the National Diagnostic Evaluation (abbreviated as END in Spanish) [20]. The END focuses on applying content from a pedagogical dimension rather than on declarative knowledge. Likewise, the act proposes that each institution take a diagnostic evaluation of its students upon admission to a teaching degree to address possible gaps with the admission profile proposed by each institution.

Since 2007, in addition to the mandatory accreditation of teaching degrees, these elements have slowed down enrollment. Nevertheless, from 2000, teaching degrees experienced an increase in their offer due to non-existent regulation and low selection criteria [21]. Specifically, establishing ITT standards addresses educational deregulation by providing universities with a minimum common action framework. It also promotes state participation in training future teachers [22] to raise the performance standards of education graduates when entering the school system [23]. Thereby, the quality of the training provided by the national school system would be higher.

The mechanisms put forward since 2007 have forced universities to enhance the quality of their ITT and change the training focus. The institutions have acknowledged a weak articulation between general teaching formation and specialty training [24]. They have also considered the pre-eminence of content over skills vital to promoting practical training [25]. The institutions also addressed new challenges such as plurality, diversity, and socioemotional elements [26]. Said needs became evident during the COVID-19 pandemic and could be reinforced from initial training. Two of the elements that take center stage are diversity and integration in the classroom. They are part of the standards, which also entails that the future teacher has links with the student and their family [27] and is prepared to comprehensively meet the learning needs and barriers.

Regarding the professional placement periods, the link with the school community must continue to be promoted, and their temporality and reflexive focus must be strengthened [28]. However, the increased interest in placements must be carried out without disregarding the theoretical aspect of teaching and disciplinary knowledge, which must be balanced to avoid the decline of the intellectual curiosity typical of an education professional in training [29].

To materialize these improvements in ITT, the institutions have implemented measures such as the development of progressive placements with the support of mentors from schools, the integration of technology in training, and the consolidation of initial training standards within the curriculum. These standards focus on topics related to teaching planning, organization of the school climate [30], and educational evaluation, which has been one of the most descended elements in the END, as well as the analysis and decision making regarding the evaluation results [31]. In this regard, the END as public policy has a paramount role, given that its results allow institutions the creation of support plans targeting the most deficient aspects of their students’ training. The END also helps with systematic improvements and, in the long run, modifies institutions’ training plans to solve the root problems that their students present in the evaluation [32].

Therefore, since 2007, higher education institutions that train teachers have sought to increase the quality of training by linking contextual elements and their educational projects with the standards of the current institutional framework [33]. However, this development must be deep and part of the institutional project, aiming beyond the degree’s accreditation. It should also provide comprehensive training to students to allow them to break with the existing correlation between the university admission test and the END. In turn, recent teacher graduates would be able to teach in any establishment, given that the trend indicates that graduates from less selective institutions seek work in schools with similar characteristics to the ones where they studied their initial education [34]. Moreover, the processes of the ITT are not yet capable of changing the representation and beliefs that graduates develop in their years of schooling [35].

## 2. Materials and Methods

The analytical methodology described by Kart and Kart [36] was used for the present review. The first step was to search for research articles on the subject. The following keywords were used (“Initial teacher training”) OR (“ITT”) OR (“Teacher training”) AND (“National diagnostic evaluation”) OR (“NDE”) AND (“University”). A total of 117 articles were identified through the 4 databases employed Redalyc (4 articles), WoS (106 articles), Scopus (0 articles) and SciELO (7 articles). For the selection of the articles, the information search was carried out on 9 March 2022. Finally, only 28 articles met the pre-established selection criteria: (a) research written in Spanish and English, (b) research that has only taken place in Chile, (c) research that uses qualitative, quantitative, or a mixed method, and (d) research that reports on the work of universities or their researchers on ITT and National Diagnostic Evaluation.

Kart and Kart’s methodology [36] was followed to answer the questions that gave rise to the research: How do Chilean universities approach ITT? Do universities consider the need for teaching student support? What is the role of the data obtained from the National Diagnostic Evaluation? What approaches have researchers adopted regarding this issue?

## 3. Results

Research by Weinstein et al. [37] aimed to analyze the opinions of school headteachers regarding the educational policies put forward during Michelle Bachelet’s government. The analysis considered evaluating issues related to quality, educational equity, government sector performance, promotion of main policies and their effects, and the public policies that should be prioritized. The research was based on the application of the survey “The voice of headteachers”. The headteachers pointed out that there is no conviction that the purposes of the Teacher Career Act are fulfilled, especially the transformation of ITT, since the accreditation processes or the admissions based on a national assessment are considered insufficient. Private sector headteachers give greater importance to teachers’ continuous training than in the public sector. In turn, the public sector seeks to improve schools’ financing or de-municipalization (the process of removing local authorities’ funding for privatization). The authors conclude that if the main priority is to improve ITT, quality could decrease when seeking continuous training. In addition, headteachers’ concern grows regarding the teaching career, which could be modified through the university’s initial training or the transformative change of teachers’ social status in Chile.

On the other hand, Lobos et al. [38] developed research that aimed to describe and analyze the causes of the low intellectual curiosity of teachers in training. This research was made up of 295 pedagogy students in primary education, of which only 10 of them participated in an interview. The study showed possible internal causes during teacher training related to an existing discrepancy between expectation and reality, low professional demand, little challenge in schools, and the teaching profession seen from a purely technical lens. In this context, low quality, little demand, and a technical standpoint of the teaching profession do not favor the commitment to intellectual curiosity development, which is essential for further learning and conceptual knowledge modelling for its transfer to the educational system, specifically in the classroom.

Furthermore, Navarro et al. [39] aimed to identify the obstacles and challenges teachers and students express about the “Integrated Pedagogical Strategies” (IPS) in the PE programs of a Chilean university. For the study, ten university professors and students participated. Semi-structured interview guidelines were used and validated by experts for information collection. The “Obstacles” and “Challenges” categories were created from the teachers’ and students’ results. “Obstacles” had the following subcategories: teaching articulation, part-time jobs, lack of communication, compulsory elements, lack of motivation, clientelistic approach, preparation and development, technique, results, incorporation of all subjects, taxation difficulties, pedagogical subjects, overwork, lack of pedagogical management, being a first-year student, evaluations, low percentage, and workload. Under the “Challenges” category, we can find: interesting and voluntary process, at the beginning, avoid emphasizing results, presentation to the authorities, IPS as everything, pedagogical management, concept knowledge, collaborative work with teachers, articulation of subjects, information process, evaluation, and stages. The authors concluded that the obstacles are related to part-time teachers’ few working hours, which generate communication issues. In turn, these hinder the development and preparation of IPS. The challenges are closely related to the transforming process that the Integrated Pedagogical Strategies should have.

The work developed by Errázuriz [40] aimed to evaluate an essay writing process by teaching degree students from the Araucanía and Los Ríos regions in Chile. The sample consisted of 205 primary teaching students that sat for an essay writing assessment. It consisted of ten dimensions and four performance criteria. The results show low spelling performance, poor counter argumentation, and use of intertextuality. Furthermore, the results led to conclude that the student’s performance did not progress significantly during their four years of teacher training. Finally, it should be noted that a proven mechanism to increase written composition performance and form communities of practice is peer writing centers. They help improve the performance of their participants, advance their writing conceptions, especially those of their tutors, and develop self-regulation, metacognition, and self-efficacy skills.

From a philosophical perspective, Mujica [41] developed an analysis of the PE curriculum in Chile. The objective was to analyze some problems around the PE and health school curriculum transition from a modern to a postmodern perspective and its place within the current social context. The author pointed out a curricular problem between school PE and ITT in universities. Schools have an objective-oriented vision, while future teachers have a competency-based vision. The author concluded that a rigorous approach to the problems raised is essential because there must be logic and coherence behind school PE. However, it currently has an approach towards health, losing the educational sense.

Additionally, López-López et al. [42] developed research to reflect on teacher training in a context of profound social transformations that have challenged the ITT in their pedagogical work. The article states that teacher training should align with these changes, giving greater value to socio-emotional competencies. These competencies are self-awareness, self-management, awareness of others, social skills, and responsible decision making. A competency that was not acquired cannot be taught. This analysis arises from the premise that the teacher must integrate knowledge and values into their teaching due to the socially constructed nature of their work in the context of economic inequalities and social vulnerability. Thus, the teacher is the mediator between knowledge and the processes of its assimilation. The article points out that this reassessment of socioemotional education should be deepened in contemporary teaching, considering its incorporation into ITT and its implementation in teaching. Regarding initial training, it should be incorporated into the curriculum of the teaching degrees, because it is a tool for developing a collective critical consciousness. Therefore, it is necessary that education, particularly teachers, does not only transfer knowledge with cognitive and emotional strategies in the classroom. For this reason, ITT from a socioemotional dimension is a challenge which should be addressed in formal and periodic instances of evaluation, together with the revision of the curriculum of teaching degrees. This is one of the weaknesses raised by the National Accreditation Commission (in Spanish CNA-Chile).

Furthermore, Matus-Castillo et al. [43] developed research to provide a current vision of initial PE teacher training in Chile from a gender perspective. It involved the participation of students and the development of this perspective in their training. The study carried out a descriptive analysis of secondary data and the analysis of documentary sources, such as databases, institutional reports, theoretical and methodological proposals, and national and international research. It identified a relevant under-representation of women in PE education in Chile. Gender stereotypes continue to be reproduced. Additionally, measures to incorporate, treat, and evaluate gender perspectives or inclusion are not present. Considering the investigative and methodological level, qualitative orientations predominate in the study of gender, inclusion, and stereotypes. Regarding the results, the analyzed documentation shows no relevant changes in female participation in the PE Program enrollment; female representation is considerably below male. On the other hand, no progress is perceived in the incorporation, evaluation, and treatment of the gender perspective in training. Finally, this study suggests that a series of questions require a prompt response and that not addressing the gender perspective poses a high risk of perpetuating gender gaps. The questions are: Does the PE teacher training influence the reproduction of gender stereotypes in lessons and school sports? Why do women make up a minority in PE studies? What would be the best strategies to incorporate and develop the gender perspective in the PE teacher’s ITT?

Tapia et al. [44] sought to identify if there was any change in the number of ICT (information and communication technology) subjects in teaching degrees between the years 2012 (n = 212) and 2018 (n = 237) in Chile. They compared curricular offerings of training programs. First, a list of the programs was drawn up, selecting only secondary school programs. Then, through the universities’ websites, the curricular offerings were reviewed, compiling 212 training programs. The ICT subjects mentioned and those contributing to the program’s graduation profile were registered, including those transversal subjects for all the degrees across institutions. Therefore, those optional or elective subjects were not included in the database. With the data, there is a positive percentage variation in the number of programs with at least one ICT subject (12.4%) in the average of ICT subjects (29.5%), in the number of programs that include two ICT subjects, and differences in the number of programs with ICT presence between disciplinary groups. Finally, the article points out that there is concern about incorporating ICT subjects in teacher training. The incorporation of ITC is very dissimilar even in programs of the same specialty offered by different institutions because it has not permeated the teacher training curriculum of higher education institutions.

In the Soto-Hernandez and Díaz [45] study, the satisfaction and perception of graduate teachers regarding their initial training at Universidad de Concepción (Los Angeles and Concepción sites) was analyzed. This research is quantitative, descriptive, and cross-sectional. For data collection, a survey was designed using two official MINEDUC (Ministry of Education) teaching documents, the Framework for Effective Teaching (FET) and the ‘Pedagogical Guiding Standards for Primary and Secondary Teaching’. The sample was 115 teachers in placement. The data were analyzed with the SPSS program version 22.0. Descriptive analyses were performed, and the Student’s *t*-test was applied to compare the results of the professors linked to the different campuses. The results showed high satisfaction of the respondents in various aspects of the ITT offered by the Universidad de Concepción. Furthermore, there were statistically significant differences in satisfaction with ITT in favor of a specific campus. The authors point out that the results of this study are not representative of the graduates of the Universidad de Concepción, nor can they be generalized to teachers from other universities. However, they constitute a new antecedent to low results in the standardized test INICIA and a good perception regarding ITT. Undoubtedly, this contradiction reflects the need to investigate the marked difference between the perception of training and the evidence of mastery of basic knowledge.

The investigation by Maldonado-Fuentes [46] describes the primary education students’ opinions from a university in the country’s center-south about using the One Minute Paper (OMP) as an evaluation and feedback mechanism. The study is descriptive, cross-sectional, and does not meet the sampling criteria of representativeness. It is not intended to generalize the results, so the author recommends making contextualized use of the conclusions. The information was collected through an ad hoc structured survey of 41 primary education students from a university in the center-south of Chile (cohort 2015). This group was chosen because they share the following characteristics: they are all regular students who have passed Curriculum and Assessment I and II and are enrolled in a subject which implements the project using OMP. Among the results of this study, it stands out that the use of OMP contributes to solving the lack of information about what students learn and how they learn at the university level. The author recommends using formative evaluation to develop specific evaluation procedures during the ITT. She also points out that the OMP is an evaluation technique that takes time, representing an additional effort for the teacher but having advantages for student evaluation and feedback based on successes and errors.

Miranda et al. [47] described the redesign process carried out by the primary education program at Universidad Católica del Norte (UCN). This process was motivated by the program’s short- and medium-term needs, such as its context and the aspects that public policy has defined as priorities for education in Chile. Action research was used as a methodology, dividing it into seven phases. The first phase focused on analyzing different documents to systematize a reference framework for decision making. In the next stage, the redesign of the training domains was defined. The final competencies were defined in the third phase, and the Graduate Profile was prepared. In phase four, with the Graduate Profile completed, the global structure of the programs was specified. The following phase continued with the creation of the ‘Intermediate or progress Profile’, and the competencies articulation of each profile was reviewed. Finally, in stage six, the Entry Profile was defined to begin the review of the consistency and progression of all the (sub)competencies declared in the three profiles for each training domain in the next phase. The necessary modifications were made to this collaborative work, and the final versions of these documents were proposed. The authors conclude that the redesign strengthened teamwork, the institution’s improvement culture, and enabled obtaining a quality product, which the National Accreditation Commission validated.

In the study developed by Sánchez-Sánchez et al. [48], the pedagogical experiences of teachers in training are explored. This deepens the topics related to teachers’ identity, or who they feel or seek to be, as well as the main obstacles, problems, or limitations teachers must face when completing their training process successfully. The methodology used is qualitative, with an interpretive case study design. In addition, semi-structured interviews were conducted as a data collection technique. The participants were 32 students from the language, mathematics, social sciences, and natural sciences specializations who carried out their placement in primary schools. Among the main results, the following stand out. First, training teachers know that teaching involves more than purely technical aspects and relies on the context. In addition, there are different images, metaphors, or constructs about the teacher’s figure. Furthermore, training teachers notice the contradictions between the technical aspects to be met and teacher autonomy.

Calisto-Alegría [49] set out to understand the process of acquiring investigative skills in the training of language and literature teachers through interactions. The method is part of the interpretive paradigm through observations and a focus group. A ‘Degree Workshop’ course comprised 10 students and a teacher and was observed for a semester. A focus group was also held to learn about students’ dynamics and subjectivities. The results show that, even though the interactions favor the acquisition of investigative skills, the student body does not have sufficient autonomy to initiate and maintain the interactions, which could affect the development of their communication and investigative skills. At the same time, the students agree that their training in investigative skills is linked to the development of attitudes. The author concludes that the ‘Degree Workshop’ must become a place of trust, responsibility, equality, and self-regulation to promote interaction, which is fundamental to the development of investigative competence.

In the research by Ayala-Perez and Joo-Nagata [50], some digital culture aspects of teaching students who have grown up with technology (millennials) were described to guide their ITT and future professional performance. The study was exploratory and contemplated the participation of teaching students. The collection of information was carried out through a survey on the students’ characteristics, cyberculture, level of software use, use of devices, and digital skills. The results show no significant differences between the groups compared. The students seem to have homogeneous characteristics regarding their approach to technology.

The study by Maureira-Cabrera et al. [51] valued the use of technological tools to develop the evaluation and co-evaluation, allowing immediate feedback in virtual environments. This study’s correlational methodological design was applied to six student groups on the educational research subject at Universidad de Chile. The study showed a significant correlation between the use of the virtual environment in the construction and application of test-type instruments and the performance results obtained by the students. They enhance their learning by incorporating the evaluation within the training process, mediated by the support of technologies.

Rodríguez-Alveal et al. [52] conducted a study on the performance of 612 mathematics teaching students from Chilean universities who sat for the 2017 National Diagnostic Evaluation. Mainly, strengths and weaknesses are analyzed around pedagogical and disciplinary knowledge and how they are related to the years of accreditation. A mixed methodology and methodological complementarity were employed, using an ad hoc database built from the information provided by MINEDUC and data from the National Accreditation Commission (CNA-Chile) (www. cnachile.cl (accessed on 2 September 2022)) regarding the years of accreditation. The study provides empirical evidence on the effect of the years of accreditation on ITT and information on skills that should be enhanced in training itineraries.

In their research, Donoso and Ruffinelli [53] reconstructed the logic implicit in the origin of the National Diagnostic Evaluation through the assessment of different actors who worked in state entities linked to the drafting process of Act 20,903. A qualitative methodology based on semi-structured interviews was used, with questions adapted to each of the six interviewees, considering their specific characteristics and roles. One of the main findings is that there is little clarity regarding the evaluation capacity of what is declared in this instrument. In addition, some basic elements for the functioning of this mechanism are absent, such as the lack of availability of key information, coordination between the different entities involved, and the responsibility of the state.

Jarpa and Becerra [54] mainly aimed to characterize the genres of pedagogical reflection written by students of two teaching programs at a Chilean university during their placement period. The methodological approach used was qualitative with a design of multiple case studies. Semi-structured interviews and focus groups were used with students and teachers of early childhood education and special education programs. The findings highlight the recognition of writing as a fundamental resource for pedagogical reflection. Progression was identified in the use of discursive genres. Critical episodes go from descriptive to argumentative elements in coherence with the complexity and progress of the Study Plan.

The research developed by Millán [55] analyzes how the demand for inclusion and cultural diversity is addressed in the ITT processes of 68 primary education degrees in Chile. For this, graduation profiles and curricular programs comprising the number of courses oriented to diversity or special educational needs were reviewed. Among its main results, it was shown that the universities in Chile present a course associated with diversity or special educational needs in isolation from their program. Additionally, 65% of the Graduate Profiles offer generic learning in diversity that does not allow for highlighting their approaches or priorities. Furthermore, the use of the word “context” predominates. The author concluded that despite the importance of formal education, it is essential to consider that each pedagogical decision has a transforming impact on the subjects’ society.

Ferrada et al. [56] explored the features of communicative competence considered essential for the initial training of language schoolteachers in Spanish and Mapudungun. They belonged to primary and university teachers aware of linguistic diversity. It took place in the central-southern Chilean regions to compare their visions of ITT and the Dialogic Model (kishu kimkelay ta che). The participatory research method was used in two sample groups: one made up of teachers who work in schools and the other of university teachers who train future teachers. The results show a considerable gap between the concept of communicative competence proposed by both groups. One is rooted in bilingualism and biculturality linked to the territory in which they work; the other emphasizes formal and procedural aspects of the dominant language.

Dockendorff et al. [57] developed a case study investigating the impact of ICT integration on mathematical visualization skills and ITT programs. GeoGebra dynamic software’s influence in promoting high school mathematics learning and its impact on teachers’ conceptions of teaching and learning mathematics is reported. This article describes how GeoGebra-based dynamic applets, designed and used in an exploratory manner, promote mathematical processes such as conjecture. It also refers to the changes experienced by future teachers in terms of the relevance acquired by dynamic visual representations in the teaching of mathematics. This study observes school routines changing when incorporating technology in the math classroom. Visualization appears as a basic competence associated with vital mathematical processes. Finally, the implications of early ICT integration in initial mathematics teacher education and their impact on technological pedagogical content knowledge development are outlined.

On the other hand, Carcamo et al. [58] aimed to recognize the social representations that primary education teaching students have regarding the parental roles assigned to families in educational matters. The research develops the representations that future teachers of a university in the Ñuble Region (Chile) have about the family and their parental roles in educational matters. Following the objective, a comprehensive interpretative study was developed using qualitative methodology. Among the most relevant findings, it was found that the roles the participant assigned to the families are located in two dimensions: on the one hand, support in the pedagogical field through finishing homework and, on the other, the setting of habits that facilitate the establishment of a favorable classroom climate for learning.

Morales-Saavedra et al. [59] present the results of an ITT experience in an early childhood education program. The research assumes that Mapuche indigenous families and communities in Chile associate knowledge with sociocultural practices. Therefore, they would allow supporting early childhood education from an intercultural educational approach with an epistemic foundation relying on parents’ and wise men’s social memory. First, the study found that in an intercultural education context, formal and informal academic opportunities must provide students with skills to relate to other cultures. Secondly, the formal curriculum in intercultural education is a bridge with the local culture. Additionally, in intercultural education, solid training in research is required. Finally, it is necessary to develop students’ skills to overcome challenges and difficulties based on inquiry, research, knowledge, and recognition of others.

Sandoval-Rubilar et al. [60] studies the social representations (SR) of the teaching profession reported by students entering the ITT in two Chilean state-run higher education institutions, Universidad del Bio and Universidad de Los Lagos. The sample comprised 834 students from the 2018 cohort from 15 teaching programs. A Likert-type scale instrument was applied and analyzed with numerical and inferential descriptive methods for the information analysis. One aspect to highlight from the conclusions is that the answers obtained were similar, independent of the specialization area. The perception and self-perception of the teacher’s SR are independent of specialization. Under this premise, it is essential to take this perception into account and it is proposed to develop a joint support plan aimed at teaching programs to work on improving their ITT. A limitation of the instrument application is highlighted due to its inability to depict a national picture accurately. The recommendation is to apply the research in different areas of the country to obtain results that are more representative of reality and thus be able to improve ITT even further.

Godoy et al. [61] presented an article that substantiates that the ITT’s teaching–learning processes in natural sciences should be enhanced through ICT, specifically in chemistry. It then takes advantage of the current generation of students using technology often. Furthermore, the coherence between the students’ entry skills and the low performance of standardized tests was reviewed, concluding that it is essential to adjust the teaching–learning processes in the ITT mediated by technologies, which necessarily implies a transformation of these teachers into agents of change. As a result, they will have the appropriate tools for their teaching in the students’ context.

Brun et al. [62] collected the results of a study on the availability and use of ICT in 46 ITT institutions in Chile implemented in 2009 as part of the OECD international project “ICT in Initial Teacher Training”. This study methodology combines both quantitative and qualitative data collection techniques. Surveys were applied to different participants in the Initial Teacher Education institutions in Chile using self-administered paper questionnaires. In addition, several case studies (mainly involving interviews and focus groups) were carried out in selected institutions according to different criteria. This work’s main quantitative statistical procedures correspond to descriptive analysis. As a conclusion, it can be highlighted that institutional contexts are generally conducive to the integration of ICT. Teachers use ICT as part of the methodology in the classroom for students, although it is only restricted to some resources. Despite this, ICT use is still limited due to the lost possibilities for innovation. The expectations regarding the pedagogical integration of ICT in Chile have not yet been fulfilled, since most students are not being taught how to use the full range of ICT resources available.

Zambrano [63] carried out a quantitative and descriptive study to quantify, describe, and analyze the lexicon of third-semester Chilean students. A total of 106 pedagogy students in the third year of their program participated. The sample comprised 60.6% female and 39.4% male subjects, aged between 20 and 22. The conclusions of this study are associated with three areas. First is applied linguistics: the results show that the centers of interest in self-regulation of learning and monitoring of the study present fewer words. Second is self-regulation of learning: this is not immediately associated with a learning process because word learning is not evoked in any center of interest. The third is ITT: categories were obtained that represent the teacher’s role in teaching, didactic resources for teaching, and external regulation of the study, rather than the representation of the categories themselves of the phases of self-regulated learning. For the author, the challenge of creating a professional teacher profile based on innovation that contributes to self-regulated learning emerges.

On the other hand, Castro-Rubilar and Navas-Martínez [64] developed non-experimental research to assess students’ perceptions in early teaching placements. A total of 164 students from the seventh semester of the primary teaching program at Universidad del Bío-Bío participated. The instrument used for the research development was created, referencing the competencies of the graduation profile of the said program. The investigation concludes that the acquisition of pedagogical knowledge related to curricular planning (class and evaluation) and curricular management activities for developing a class predominate.

## 4. Discussion

The articles explored show the topics that have interested researchers and universities about ITT in Chile. These deal with diverse aspects grouped mainly in competencies to be developed: use of ICT and methodologies and evaluation. To a lesser extent, the following topics are addressed: gender perspective; inclusion and diversity; opinions on training and the teaching profession; curriculum; and curricular redesign experiences.

The topics that group a greater number of articles refer to “competencies to be developed”. These are closely linked to the training model that Chilean universities have implemented in recent years. These institutions declare that their training is competency-based. Thus, the teaching and learning process aims for students to acquire abilities, knowledge, and skills using procedures or attitudes necessary to improve their performance and achieve the goals outlined by the higher education institution for a certain program or degree [65]. Tobón [66] explains that competencies allow the integration of different knowledge such as knowing how to be, do, know, and live together. They allow carrying out activities or solving problems to contribute to personal development, the construction and strengthening of the social fabric, the continuous search for sustainable economic–business development, and the care and protection of the environment and living species.

In this line, the research reviewed investigates the acquisition of specific skills considered fundamental in the training of a future teacher [67], including intellectual curiosity, writing, research, reflection, and communication skills. These skills are approached from their application in different contexts or the students’ perceptions. The researchers agree that there are difficulties in acquiring certain skills, so they suggest that training programs and universities develop strategies to address these deficiencies and provide future teachers with the necessary skills for a professional performance.

Another topic of interest is the study of ICT and its incorporation into ITT’s teaching and learning processes. ICT enables the convergence of the different technological tools that produce, receive, store, share, access, and process information, which is presented through images, texts, and sounds, among other mediums [68]. Their usefulness allows access to information and incorporates different methodologies to bring knowledge closer to people. In this context, universities are responsible for providing opportunities for the use of ICT, since its adoption will provide a context for digitization and will open possibilities for students [69]. The analyzed research investigates the incorporation of ICT in the teacher training curriculum. Others have worked on the relationship of digital culture with students entering the university. Finally, a group has analyzed the use of ICT in teaching different disciplines. These investigations agree that the use of ICT is favorable, since it contributes to teaching through the incorporation of different methodologies and learning, considering that current generations are closely related to ICT. Although the investigations show increased ICT-related subjects’ presence in the national curriculum, they conclude that these should have an essential role in teacher training. Moreover, the experience left by the COVID-19 pandemic forced institutions to teach virtually worldwide [70].

Another topic that has brought researchers together is related to methodologies and evaluation, both key elements in the teaching and learning processes. It is currently hoped that methodologies favor active participation and cooperative work relationships to develop skills such as problem-solving and increase creativity and critical reflection in students [71]. For this reason, these have been investigated; however, there are not many subject-related investigations associated, being a niche to explore in teacher training in Chile. Regarding evaluation, this is a complex and systematic process that allows collecting information to make judgments and decisions regarding student learning and development [72]. The investigations work on this theme from two different angles. One is the standardized evaluations for future teachers or END and the results’ implications. The second angle is the analysis of strategies that allow evaluation development in different contexts. The research associated with the END gives an account of the competencies achieved and the factors that influence the results obtained in this evaluation. Those referring to evaluation strategies show how evaluation can be developed with different tools to make the process more efficient.

### 4.1. Limitations

Within the limitations of this study, it is possible to point out that the review presented is only based on articles published in Spanish, excluding those published in another language, so there could be other topics that have not been addressed in this study.

### 4.2. Practical Implications

ITT is a process born from a conception related to improving the quality of education. Under this view, primary and secondary education should significantly improve in different school contexts. In theory, it is expected that, through law, there will be an impact not only at the level of higher education but also at the level of initial school education through a transfer that teachers in training should make once they start teaching.

In this way, it is expected that with the incorporation of the requirements of the law and, in particular, with the application of the National Diagnostic Evaluation—applied one year before graduation—the students’ weakest theoretical and practical knowledge will be detected proactively. Thus, higher education institutions can make decisions in this regard, but how can that knowledge be quantified or measured? Is knowing these data a year before graduation enough? These questions open new work paths and reflection around ITT and how it is considered in Chilean higher education. The law also establishes new access routes to teaching programs, such as Access Programs [73], which try to provide students who wish to study teaching with the minimum competencies. This could improve performance not only in the END but also contribute to ITT.

On the other hand, training in the school system must be improved, just as higher education must further strengthen its levelling programs. Evidence continues to show a strong correlation between the scores for access to higher education and END results, generating the question of why the institutions are not yet capable of breaking this relationship. Generally speaking, this is a complex situation marked by the first years of schooling, unfortunately associated with the families’ economic investment in their children’s education.

Higher education institutions must direct their work towards incorporating regulation and adapting training programs to standards to achieve curricular alignment.

### 4.3. Future Directions

Based on the findings obtained in this review, it would be relevant for higher education institutions to promote research with a view to the new challenges that emerge in the educational system, such as inclusion, diversity, and migration, among others. Gathering information on these areas could support decision making regarding ITT in Chile. In this way, patterns, variables, or results could be detected. They could serve as support in the first years of teacher training to break the sequentiality of the results that show the shortcomings of school and university training. It is also important that public policy can advance in the definition of certain core subjects that should be in all teaching training curricula. This would ensure a common knowledge foundation for future teachers. Although the standards are guiding, they are always personalized by each institution based on their needs, identity, and realities. In order to further the system’s quality, some key elements must be standardized. The accreditation processes do not necessarily evaluate those key aspects because an important percentage of a program’s accreditation relies on institutional and economic aspects rather than training itself.

Finally, the research must be carried out in other areas of knowledge since many of the studies analyzed correspond to PE. Therefore, it would be interesting to start studying the phenomena studied in other areas.

## 5. Conclusions

Chilean legislation currently establishes guidelines, agreements, and duties that regulate ITT from the beginning of the teaching program, during their studies, and across the teaching career. Through this review, some aspects were evidenced that possibly significantly impact teacher training and should be considered by higher education institutions and national educational policy. Firstly, the opinions of headteachers point to the importance of accompanying teachers throughout their careers, which would allow their comprehensive development. Secondly, the importance of levelling competencies or skills was mentioned. For example, ICT, reading comprehension, and others should be acquired in secondary education. These competencies should be deepened in the ITT due to the impact these have on teaching. Thirdly, the scope of public policies in primary and secondary education and how these could affect ITT’s quality was discussed. Finally, the social representations around the profession, the new generations of students, and the student’s characteristics and perceptions of the teaching career were mentioned. All these elements provide us with evidence of the decisions that must be made regarding the teaching profession and the improvement of initial training.

On the other hand, it is evident that the support for students in ITT is still under development, since no studies show how the university system integrally develops this support. Although END data have been discussed in some studies, there is still no evidence of how the problems detected in the initial training are being corrected. There is then a challenge around using the information and how improvement plans are proposed within educational institutions.

The issues addressed in the different investigations highlight the need to work on new issues related to ITT. In addition, discussing those already recurrent problems in the Chilean educational system would help to achieve adequate training relevant to new contexts and capable of responding to the needs of a changing society

## Data Availability

Not applicable.
